# Supplementary material for: Norovirus infection causes acute self-resolving diarrhea in wild-type neonatal mice
Source: Nat Commun. 2020 Jun 11;11:2968. doi: 10.1038/s41467-020-16798-1 (PMC7289885; doi:10.1038/s41467-020-16798-1)
Supplement: Supplementary file 1 — Supplementary Information [file 41467_2020_16798_MOESM1_ESM.pdf]

Supplementary Information

Norovirus infection causes acute self-resolving diarrhea in wild-type neonatal mice

Roth and Helm et al.

Supplementary Figure 1

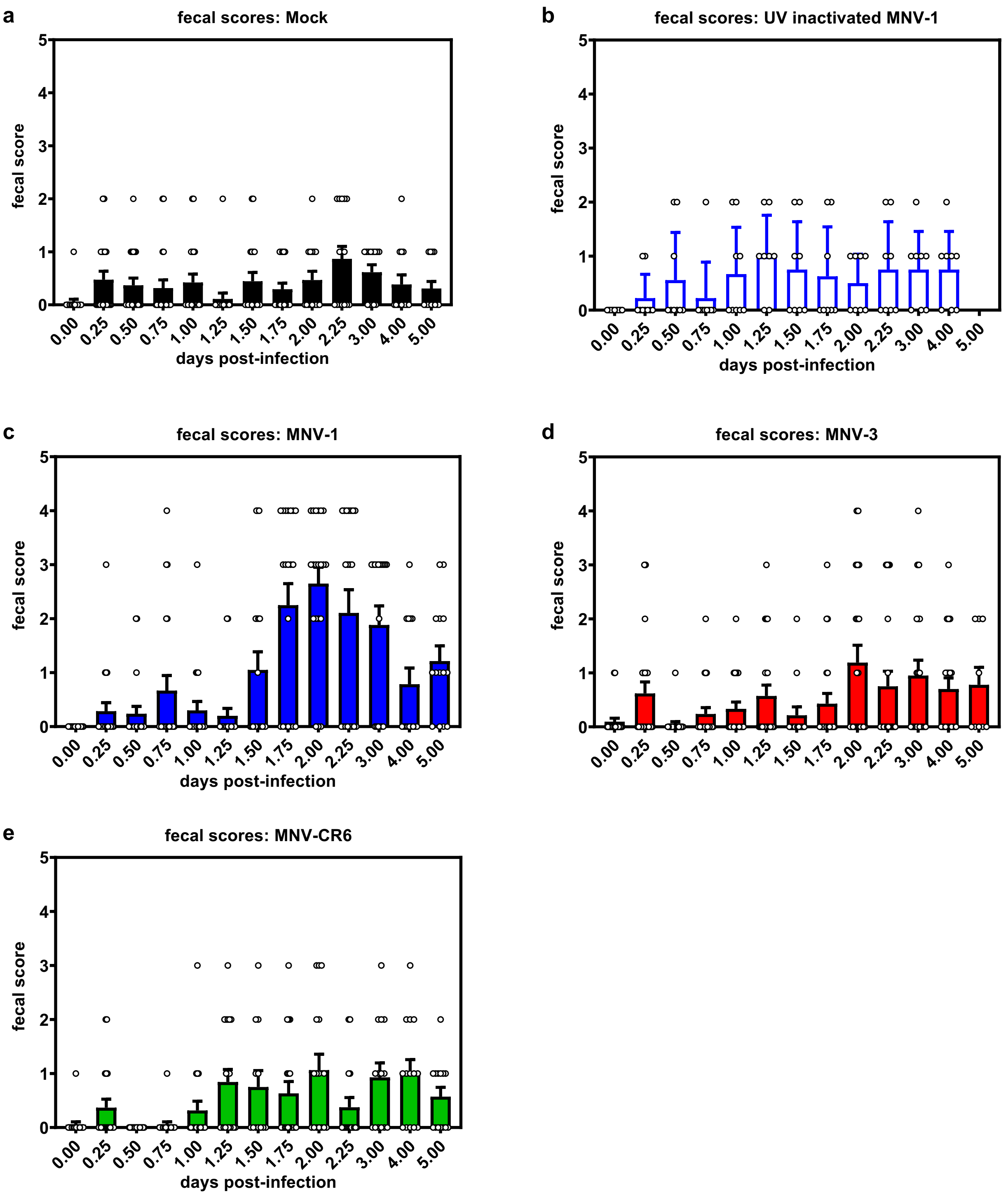

Supplementary Figure 1. Distribution of fecal scores. The data presented in Fig. 1d are shown here as a bar graph with scores for individual mice represented by dots for a) mock (n = 19), b) UV inactivated MNV-1 (n = 9), c) MNV-1 (n = 21), d) MNV-3 (n = 21), and e) MNV-CR6 (n = 19). Error bars denote standard errors of mean in all figures. Source data are provided as a Source Data file.

Supplementary Figure 2

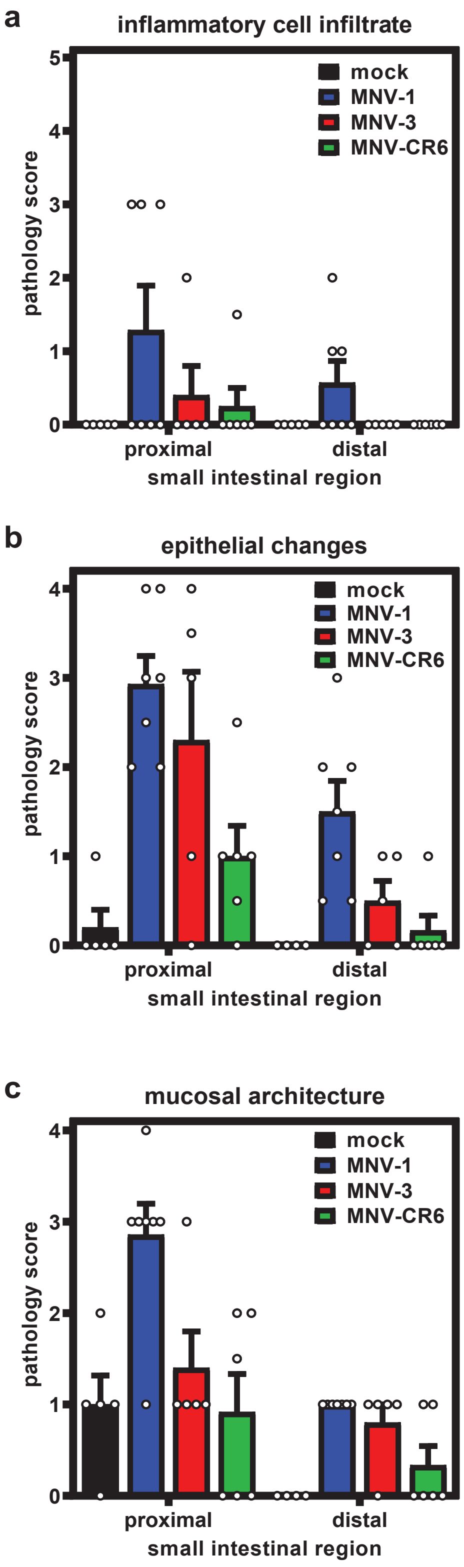

Supplementary Figure 2. Pathology scores for each scoring criteria. Small intestinal sections collected from neonates infected with 10^8 TCID50 units of MNV-1 (blue), MNV-3 (red), MNV-CR6 (green), or mock inoculum (black) at 2 dpi (n = 5 for mock, n = 7 for MNV-1, n = 5 for MNV-3, n = 6 for MNV-CR6) were stained with hematoxylin and eosin. Sections were scored blindly by an animal veterinarian for pathological changes in regards to inflammatory cell infiltrate (a), epithelial changes (b), and mucosal architecture (c). Source data are provided as a Source Data file.
